# Prevalence of co-occurring conditions among youths receiving treatment with primary anxiety, ADHD, or depressive disorder diagnoses

**DOI:** 10.3389/frcha.2024.1340480

**Published:** 2024-04-16

**Authors:** Orrin D. Ware, Lisa D. Zerden, Jacquelynn F. Duron, Yanfeng Xu, Lauren P. McCarthy, Sarah Verbiest, Jenny Afkinich, Qiana Brown, Denise Yookong Williams, Trenette Goings

**Affiliations:** ^1^ School of Social Work, University of North Carolina at Chapel Hill, Chapel Hill, NC, United States; ^2^ School of Social Work, Rutgers University, New Brunswick, NJ, United States; ^3^ College of Social Work, University of South Carolina, Columbia, SC, United States; ^4^ School of Medicine, University of Colorado Anschutz Medical Campus, Aurora, CO, United States

**Keywords:** attention-deficit/hyperactivity disorder, anxiety, community mental health center, depression, substance use, youth

## Abstract

**Introduction:**

Anxiety disorders, depressive disorders, and attention-deficit/hyperactivity disorder (ADHD) are some of the most common conditions that youths (<18 years old) receive mental health treatment for. These conditions are associated with high-risk substance use or substance use disorders (SUDs). This study sought to identify the proportion of youths (<18 years old) with anxiety disorders, depressive disorders, or ADHD as a primary diagnosis in community mental health centers (CMHCs) having co-occurring high-risk substance use or a SUD.

**Methods:**

Analysis included binary logistic regression models using the Mental Health Client-Level Data 2017–2019 datasets which contains annual cross-sectional administrative data from mental health treatment facilities. The final sample included *n* = 458,888 youths with an anxiety disorder as a primary diagnosis, *n* = 570,388 youths with a depressive disorder as a primary diagnosis, and *n* = 945,277 youths with ADHD as a primary diagnosis.

**Results:**

In the subsample with anxiety as a primary diagnosis, approximately 5% of youth had high-risk substance use or a SUD. Approximately 10% of youth with depression as a primary diagnosis had high-risk substance use or a SUD. Among youth with ADHD as a primary diagnosis, 5% had high-risk substance use or a SUD. Odds of having a co-occurring high-risk substance use or SUD differed based on the youth’s age, race and ethnicity, gender, and other mental health diagnoses.

**Conclusions:**

Effective care for this high-need youth population at CMHCs will require mental health clinicians to possess knowledge and skills related to substance use treatment.

## 1 Introduction

Anxiety disorders, depressive disorders, and attention-deficit/hyperactivity disorder (ADHD) are among the most common mental health conditions impacting children and adolescents. Among U.S. individuals aged 3–17 years, 9.4% have ever been diagnosed with anxiety, 4.4% have ever been diagnosed with depression, and 9.8% have ever been diagnosed with ADHD (1). The gravity of these proportions is underlined by evidence that anxiety disorders, depressive disorders, and ADHD can impact several social, economic, and developmental factors, including academic attainment, interpersonal relationships, and quality of life (2–8). Yet, just 10% of children and adolescents ages 3–17 years old received mental health treatment for any mental or behavioral health disorder (1, 5), highlighting a major service shortfall with concerning implications for this population as they age into young adulthood.

Evidence shows that increasing mental health treatment engagement, including technology-delivered interventions, is associated with improvements in mental health (9, 10). Mental health treatment settings for youths include psychiatric hospitals, residential treatment centers, and community mental health centers (CMHCs). CMHCs are entities licensed by a state or governing licensing board that provide specialized services for mental health symptoms and disorders (11).

Although mental health facilities such as CMHCs specialize in mental health treatment, substance use disorders (SUDs) often co-occur with mental health disorders (12–14). Approximately one in four persons ages 12–17 years had either a major depressive episode or a SUD in 2021 (14). Further, nearly one million individuals ages 12–17 years had a co-occurring major depressive episode and a SUD (14). Also, individuals with ADHD are more likely to develop a SUD than individuals without ADHD (15). These findings underscore the need for clinicians to comprehensively and concurrently treat mental health disorders and SUD as comprehensive, integrated treatment is seen as a best practice (16, 17). Further, evidence suggests that integrated models of treating co-occurring conditions are more cost-effective and have better clinical outcomes compared to non-integrated treatments for co-occurring disorders (18). Less burden is placed on individuals with co-occurring disorders when integrated care is provided by the same treatment team instead of siloed by separate providers (16, 18). Although integrated treatment is seen as best practice (16), several provider-level barriers to implementing these services exist, including training barriers, limited time to provide services to individuals, concerns related to patient satisfaction, institutional policies, lack of staff, and limited resources (19). While there may be a desire and willingness to provide integrated treatment for co-occurring disorders, several legal, institutional, training, and resource-level barriers increase the difficulty of many clinicians to do so (19), further limiting the availability of these services for individuals in need of co-occurring treatment.

Many mental health treatment facilities are unequipped to provide SUD treatment (20) or treatment to persons with high-risk substance use, operationally defined as using substances in a way that increases the risk of experiencing harmful outcomes (21). It is critical to understand the prevalence of co-occurring high-risk substance use or SUD alongside other co-occurring mental health disorders among youths with anxiety disorders, depressive disorders, or ADHD using real-world national and state level administrative data from mental health facilities.

Evidence suggests that the prevalence of co-occurring disorders varies by demographic profiles of youths (22–24). One study examining the prevalence of co-occurring major depressive episodes and co-occurring SUD among youths found that girls, compared to boys, had a higher prevalence of these co-occurring conditions (22). However, while girls were more likely than boys to receive treatment for a major depressive episode only, they were not more likely to receive treatment for the SUD only or treatment for both the major depressive episode and SUD (22). That same study found differences based on age, such as older youths were more likely to have both conditions compared to younger youths (22). Differences based on race and ethnicity were also identified, such as Hispanic individuals and those classified as “Other” race and ethnicity were more likely to have these co-occurring conditions than White youths, whereas Black youths were less likely to have these co-occurring conditions (22). Differences by race and ethnicity in co-occurring disorders have also been identified by other studies (25). In conclusion, some demographic and diagnostic profiles may have stronger associations with high-risk substance use and SUD. With this consideration, we leveraged a large dataset of administrative data to examine demographic characteristics and clinical diagnoses among youths receiving treatment at CMHCs.

To this end, the present study posed four research questions: (1) What proportion of children and adolescents with anxiety disorders as a primary diagnosis in CMHCs have co-occurring high-risk substance use or a SUD?; (2) What proportion of children and adolescents with depressive disorders as a primary diagnosis in CMHCs have co-occurring high-risk substance use or a SUD?; (3) What proportion of children and adolescents with ADHD as a primary diagnosis in CMHCs have co-occurring high-risk substance use or a SUD?; and (4) What are the proportions of other mental health disorder diagnoses among children and adolescents with an anxiety disorder, depressive disorder, or ADHD as their primary diagnosis (26). Answering these research questions will have the potential to provide greater epidemiological insight into co-occurring mental health conditions among youths receiving mental health treatment.

## 2 Material and methods

### 2.1 Data source and sample selection

This exploratory study examined a large sample of persons ≤17 years old with an anxiety disorder, depressive disorder, or ADHD as their primary diagnosis who received treatment from a CMHC in the United States between 2017 and 2019. We examined the proportion of youths with high-risk substance use or SUD in this sample. We also examined the proportion of other co-diagnosed mental health disorders in this sample. To answer our research questions, we used three years of data from the Mental Health Client-Level Data (MH-CLD) dataset. Provided annually by SAMHSA, this publicly available de-identified cross-sectional dataset contains data from individuals who received treatment within a given year in the United States from mental health providers who report their data to a state or governing body. The three years of data that we used were the MH-CLD 2017–2019 for data received and processed by SAMHSA to July 2020 (27–29). The unit of analysis is individuals receiving mental health treatment within a given year. Notably, the data are de-identified, and it is not possible to track individuals across age, years, or states; therefore, individuals may be captured in the merged dataset multiple times.

After combining these three datasets, the total number of cases included 18,290,012 individuals, which was further distilled using the following selection criteria: (1) received treatment from any CMHC; (2) had an anxiety disorder, depressive disorder, or ADHD as the primary diagnosis; (3) were minors (i.e., ≤17 years old); and (4) received treatment in one of the four regions of the United States, which resulted in a sample of 2,252,281 cases. Missing value analyses were conducted to examine if the variables included in the multivariate analyses were missing completely at random (MCAR). The data were deemed MCAR based on Little's (30) MCAR test (finding set at *p* > .05), and a final analytic sample of *N *= 1,974,553 was used for our analyses after excluding cases with missing values. We further divided this final analytic sample into three subsamples of youths: those with an anxiety disorder as a primary diagnosis (*n *= 458,888), those with a depressive disorder as a primary diagnosis (*n *= 570,388), and those with ADHD as a primary diagnosis (*n* = 945,277).

### 2.2 Measures

Measures in our study included: (1) region, (2) age, (3) gender, (4) race and ethnicity, (5) anxiety disorder diagnosis, (6) ADHD diagnosis, (7) conduct disorder diagnosis, (8) depressive disorder diagnosis, (9) oppositional defiant disorder diagnosis, (10) trauma- or stressor-related disorder, (11) another mental health disorder, and (12) high-risk substance use or SUD.

#### 2.2.1 Region

This variable identifies in which of the four U.S. census regions treatment was received (31). This variable was used as a descriptive variable.

#### 2.2.2 Age

Age in years is a categorical instead of a continuous variable in this dataset with three values: 0–11 years old, 12–14 years old, and 15–17 years old. This variable includes the actual categories provided in the MH-CLD. This variable was used as an independent variable in the multivariate analyses. For the multivariate analyses, the 15–17 years age group was the reference.

#### 2.2.3 Gender

Gender included two values in the MH-CLD dataset: boys and girls (male and female in the dataset). This variable was used as an independent variable in the multivariate analyses. Within this dataset, gender was a binary variable and did not include non-binary persons. For the multivariate analyses, girls were the reference group.

#### 2.2.4 Race and ethnicity

To account for the unique cultural experiences of Hispanic individuals, regardless of racial identity, race and ethnicity were combined. This variable was created by combining two variables found in the dataset, one which corresponds to race and the other which corresponds to ethnicity. Combining these two variables provided four values: Black, Hispanic or Latino of any race, White, and Another Race or Ethnicity. Several groups were combined into the “Another Race or Ethnicity “ value due to small cell sizes. This variable was used as an independent variable in the multivariate analyses, with White as the reference group.

#### 2.2.5 Mental health disorder diagnoses

This category includes seven non-mutually exclusive variables that identify in the individual was diagnosed with conditions. These variables were binary with Yes/No values and include: (1) anxiety disorder diagnosis, (2) ADHD diagnosis, (3) conduct disorder diagnosis, (4) depressive disorder diagnosis, (5) oppositional defiant disorder diagnosis, (6) trauma- or stressor-related disorder diagnosis, and (7) another mental health disorder diagnosis. These variables were based on the presence of these diagnoses as the individual's first, second, or third mental health diagnosis (there are a maximum of three diagnoses in the dataset). These seven variables were used as independent variables in the multivariate analyses, with no as the reference group. Variables were only added to the multivariate models if they were not the individual's primary diagnosis. The variable “another mental health disorder diagnosis” included any of the following diagnoses from the dataset, “other disorders/conditions” “bipolar disorders” “delirium, dementia”, “personality disorders”, “pervasive developmental disorders”, and “schizophrenia or other psychotic disorders” of which the last five disorders had small cell sizes in the total sample ranging from 0.0% to 1.6%. These seven variables were used as independent variables in the multivariate analyses, with no as the reference group.

#### 2.2.6 High-risk substance use or SUD

This variable served as the dependent variable for this study. This variable was created by combining two variables, one which identified if an individual had a SUD diagnosis and another which identified if the individual had a substance use problem. SUD was a binary variable in the dataset, affirming or denying the presence of a SUD. The substance use problem variable was a binary variable in the dataset that affirmed or denied the following statement: “Specifies the client's substance use problem based on a substance use diagnosis and/or using other identification method such as substance use screening results, enrollment in a substance use program, substance use survey, service claims information, or other related sources of data” (27–29). If either variable received an affirmative answer, the individual was coded as having high-risk substance use or SUD. Individuals were coded as not having high-risk substance use or SUD if neither variable was coded affirmatively. A study exploring trauma- and stressor-related disorders among adults in psychiatric hospital settings included this variable (32).

### 2.3 Statistical analyses

Version 28 of IBM SPSS (IBM Corp, 2021) was used to analyze the data. Univariate statistics were used to describe the full study sample, the subsample with a primary anxiety disorder diagnosis, the subsample with a primary depressive disorder diagnosis, and the subsample with a primary ADHD diagnosis. Three multivariate logistic regression models were conducted to examine the presence of high-risk substance use or SUD (32) among samples based on: (1) primary diagnosis: anxiety disorder (2) primary diagnosis: depressive disorder, and (3) primary diagnosis: ADHD. Due to the large sample size, an *a priori* significance level of *p* < .001 was used (33). State-level data of the percentages of high-risk substance use and SUD in the full sample and all three subsamples (Subsample 1. Children and adolescents with anxiety as a primary diagnosis; Subsample 2. Children and adolescents with depression as a primary diagnosis; Subsample 3. Children and adolescents with ADHD as a primary diagnosis) were plotted onto a map of the U.S. using the open-source software package usmap (34) to create the figures in R Version 4.2.1 [R (35)].

## 3 Results

### 3.1 Sample characteristics

**Figure 1:**
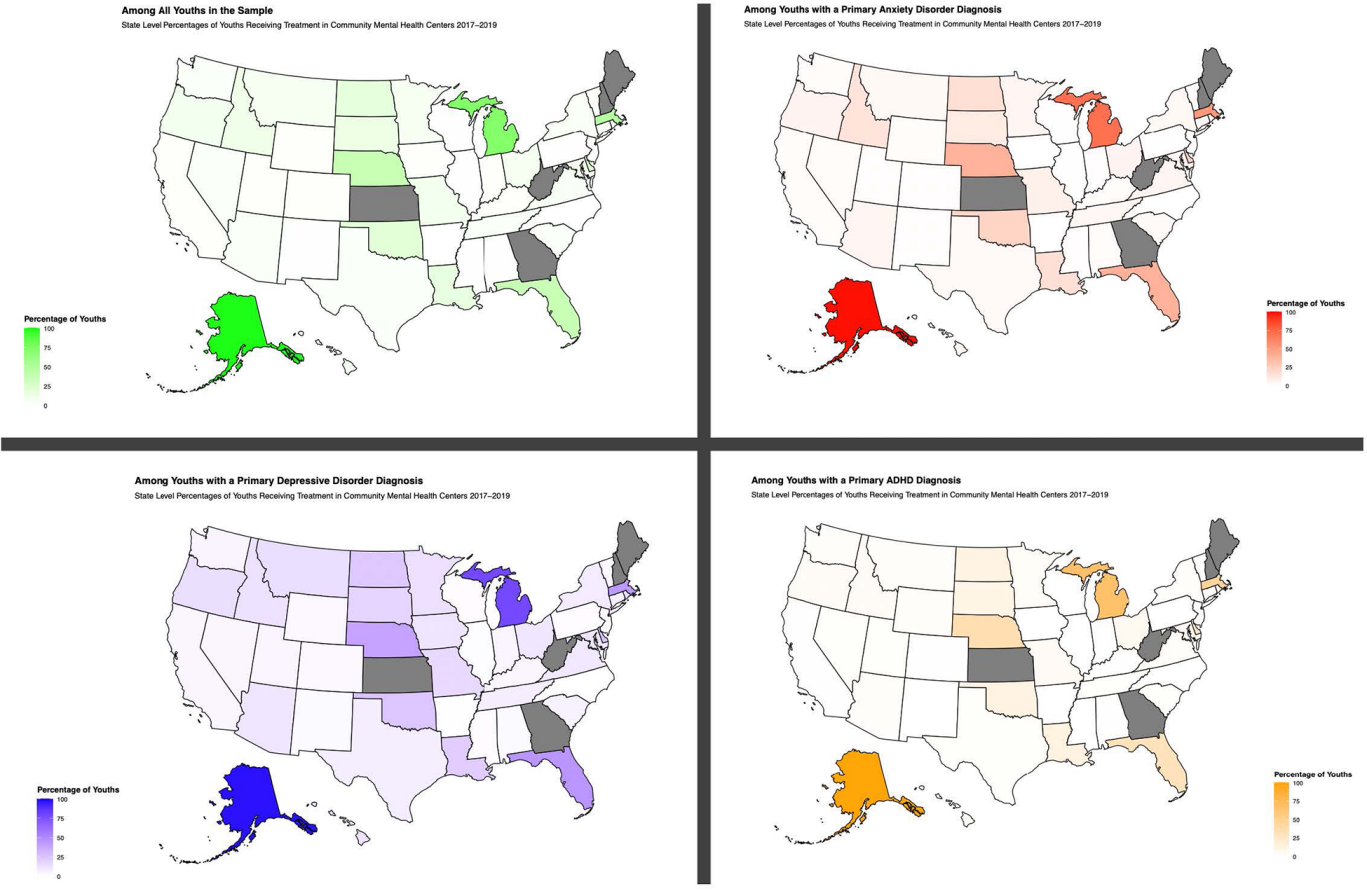
Co-occurring high-risk substance use or substance use disorders among youths receiving mental health treatment. Gray states did not have data.

Table 1 provides descriptive data about the full sample and three subsamples. Most of the sample were boys (*n *= 1,107,801; 56.1%) and had an ADHD diagnosis (*n *= 1,044,488; 52.9%). Of the full sample, approximately 6.5% of the sample had high-risk substance use or a SUD. Figure 1 provides state-level percentages of high-risk substance use or SUD among the full sample and subsamples. The full list of state-level percentages is provided in Supplementary Table S1.

**Table 1:** Characteristics of the sample.

Characteristics	Full study sample *N* (%)	Subsample with anxiety as the primary diagnosis *n* (%)	Subsample with depression as the primary diagnosis *n* (%)	Subsample with Attention Deficit/Hyperactivity Disorder (ADHD) as the primary diagnosis *n* (%)
Sample size	1,974,553 (100.0%)	458,888 (100.0%)	570,388 (100.0%)	945,277 (100.0%)
Region				
Midwest	509,899 (25.8%)	125,837 (27.4%)	133,658 (23.4%)	250,404 (26.5%)
Northeast	280,439 (14.2%)	50,000 (10.9%)	56,839 (10.0%)	173,600 (18.4%)
South	611,417 (31.0%)	93,871 (20.5%)	161,407 (28.3%)	356,139 (37.7%)
West	572,798 (29.0%)	189,180 (41.2%)	218,484 (38.3%)	165,134 (17.5%)
Age				
0–11 years	872,715 (44.2%)	208,335 (45.4%)	91,455 (16.0%)	572,925 (60.6%)
12–14 years	533,562 (27.0%)	117,313 (25.6%)	187,985 (33.0%)	228,264 (24.1%)
15–17 years	568,276 (28.8%)	133,240 (29.0%)	290,948 (51.0%)	144,088 (15.2%)
Gender				
Boys	1,107,801 (56.1%)	209,676 (45.7%)	205,197 (36.0%)	692,928 (73.3%)
Girls	866,752 (43.9%)	249,212 (54.3%)	365,191 (64.0%)	252,349 (26.7%)
Race and ethnicity				
Black	382,377 (19.4%)	46,311 (10.1%)	80,372 (14.1%)	255,694 (27.0%)
Hispanic or Latino any race	443,521 (22.5%)	109,171 (23.8%)	161,443 (28.3%)	172,907 (18.3%)
White	1,011,663 (51.2%)	267,586 (58.3%)	284,181 (49.8%)	459,896 (48.7%)
Another race or ethnicity	136,992 (6.9%)	35,820 (7.8%)	44,392 (7.8%)	56,780 (6.0%)
Mental health disorder diagnosis^a^				
Anxiety dis.^b^	618,846 (31.3%)	458,888 (100.0%)	98,161 (17.2%)	61,797 (6.5%)
ADHD^c^	1,044,488 (52.9%)	51,962 (11.3%)	47,249 (8.3%)	945,277 (100.0%)
Conduct dis.	50,973 (2.6%)	7,551 (1.6%)	8,954 (1.6%)	34,468 (3.6%)
Depressive dis.	636,896 (32.3%)	38,403 (8.4%)	570,388 (100.0%)	28,105 (3.0%)
Oppositional defiant dis.	156,371 (7.9%)	12,788 (2.8%)	20,134 (3.5%)	123,449 (13.1%)
Trauma-or stressor dis.^d^	131,574 (6.7%)	30,518 (6.7%)	44,180 (7.7%)	56,876 (6.0%)
Another mental health dis.	177,955 (9.0%)	39,872 (8.7%)	34,663 (6.1%)	103,420 (10.9%)
High-risk substance use or SUD^e^				
Yes	128,924 (6.5%)	23,846 (5.2%)	55,944 (9.8%)	49,134 (5.2%)
No	1,845,629 (93.5%)	435,042 (94.8%)	514,444 (90.2%)	896,143 (94.8%)

Percents are column percents.

Some variables may not equal 100.0% due to rounding.

^a^ These variables are not mutually exclusive.

^b^ Dis., disorder.

^c^ ADHD, attention deficit/hyperactivity disorder.

^d^ Trauma- or stressor related disorder.

^e^ SUD, substance use disorder.

#### 3.1.1 Youths with anxiety as the primary diagnosis subsample

Table 2 shows the results among youths with an anxiety disorder as their primary diagnosis. Compared to youths aged 15–17 years old, those who were 11 years old or younger [AOR = 0.285; 95% CI = (0.275, 0.294)] and 12–14 years old [AOR = 0.435; 95% CI = (0.421, 0.450)] had lower odds of co-occurring high-risk substance use or a SUD. Boys [AOR = 1.173; 95% CI = (1.142, 1.205)] had greater odds of the dependent variable co-occurring high-risk substance use or a SUD compared to girls. Whereas youths who were Hispanic or Latino of any race [AOR = 0.729; 95% CI = (0.703, 0.756)] had lower odds, youths who were of “Another Race or Ethnicity” [AOR = 1.577; 95% CI = (1.512, 1.644)] had greater odds of co-occurring high-risk substance use or a SUD compared to White youths. As seen in Table 2, conduct, depressive, oppositional defiant, trauma- or stressor-related, and other mental health disorder diagnoses were associated with high-risk substance use or a SUD compared to the absence of any of these conditions.

**Table 2:** Logistic regression model examining having high-risk substance Use or a substance Use disorder Among the subsample with an anxiety disorder as a primary diagnosis *n* = 458,888.

Variable	Adjusted odds ratio	95% CI	*p*
Age (Ref: 15–17 years)			
0–11 years	0.285	[0.275, 0.294]	<.001
12–14 years	0.435	[0.421, 0.450]	<.001
Gender (Ref: Girls)			
Boys	1.173	[1.142, 1.205]	<.001
Race and ethnicity (Ref: white)			
Black	1.062	[1.015, 1.110]	.008
Hispanic or Latino any race	0.729	[0.703, 0.756]	<.001
Another race or ethnicity	1.577	[1.512, 1.644]	<.001
Attention deficit/hyperactivity disorder diagnosis (Ref: No)			
Yes	0.976	[0.934, 1.019]	.261
Conduct disorder diagnosis (Ref: No)			
Yes	1.745	[1.599, 1.906]	<.001
Depressive disorder diagnosis (Ref: No)			
Yes	1.597	[1.538, 1.658]	<.001
Oppositional defiant disorder diagnosis (Ref: No)			
Yes	2.155	[2.023, 2.295]	<.001
Trauma- or stressor-related disorder diagnosis (Ref: No)			
Yes	1.309	[1.247, 1.373]	<.001
Other mental health diagnosis (Ref: No)			
Yes	1.280	[1.227, 1.336]	<.001

*Due to the large sample size, *p* < .001 was deemed statistically significant.

#### 3.1.2 Youths with a depressive disorder as the primary diagnosis subsample

Table 2 shows the results among youths with a depressive disorder as a primary diagnosis. Compared to youths who were 15–17 years old, those who were 11 years old or younger [AOR = 0.323; 95% CI = (0.312, 0.333)] and those who were 12–14 years old [AOR = 0.544; 95% CI = (0.533, 0.556)] had lower odds of co-occurring high-risk substance use or a SUD. Boys [AOR = 1.227; 95% CI = (1.205, 1.250)] had greater odds of co-occurring high-risk substance use or a SUD compared to girls. Youths who were Black [AOR = 0.922; 95% CI = (0.898, 0.947)] and youths who were Hispanic or Latino of any race [AOR = 0.690; 95% CI = (0.674, 0.705)] had lower odds of having high-risk substance use or a SUD than White youths. However, youths who were of “Another Race or Ethnicity” [AOR = 1.419; 95% CI = (1.378, 1.461)] had greater odds compared to White youths. As seen in Table 3, conduct, oppositional defiant, trauma- or stressor-related, and other mental health disorder diagnoses were associated with high-risk substance use or a SUD compared to the absence of any of these conditions.

**Table 3:** Logistic regression model examining having high-risk substance use or a substance use disorder Among the subsample with a depressive disorder as the primary diagnosis *n* = 570,388.

Variable	Adjusted odds ratio	95% CI	*p*
Age (Ref: 15–17 years)			
0–11 years	0.323	[0.312, 0.333]	<.001
12–14 years	0.544	[0.533, 0.556]	<.001
Gender (Ref: Girls)			
Boys	1.227	[1.205, 1.250]	<.001
Race and ethnicity (Ref: white)			
Black	0.922	[0.898, 0.947]	<.001
Hispanic or Latino any race	0.690	[0.674, 0.705]	<.001
Another race or ethnicity	1.419	[1.378, 1.461]	<.001
Anxiety disorder diagnosis (Ref: No)			
Yes	0.996	[0.973, 1.019]	.715
Attention deficit/hyperactivity disorder diagnosis (Ref: No)			
Yes	0.971	[0.940, 1.003]	.078
Conduct disorder diagnosis (Ref: No)			
Yes	2.086	[1.972, 2.207]	<.001
Oppositional defiant disorder diagnosis (Ref: No)			
Yes	1.878	[1.805, 1.954]	<.001
Trauma- or stressor-related disorder diagnosis (Ref: No)			
Yes	1.416	[1.375, 1.458]	<.001
Other mental health disorder diagnosis (Ref: No)			
Yes	1.317	[1.274, 1.361]	<.001

*Due to the large sample size, *p* < .001 was deemed statistically significant.

#### 3.1.3 Youths with attention-deficit/hyperactivity disorder as the primary diagnosis subsample

Table 4 shows the results among youths with ADHD as a primary diagnosis. Compared to youths who were 15–17 years old, those who were 11 years old or younger [AOR = 0.392; 95% CI = (0.383, 0.401)] and those who were 12–14 years old [AOR = 0.518; 95% CI = (0.505, 0.531)] had lower odds of co-occurring high-risk substance use or a SUD. Boys [AOR = 1.125; 95% CI = (1.101, 1.149)] had greater odds of co-occurring high-risk substance use or a SUD compared to girls. Youths who were Hispanic or Latino of any race [AOR = 0.726; (0.705, 0.747)] had lower odds, and those who were of “Another Race or Ethnicity” [AOR = 1.976; 95% CI = (1.916, 2.038)] had greater odds of having co-occurring high-risk substance use or a SUD compared to White youths. As seen in Table 4, conduct, depressive, oppositional defiant, trauma- or stressor-related, and other mental health disorder diagnoses were associated with high-risk substance use or a SUD compared to the absence of any of these conditions.

**Table 4:** Logistic regression model examining having high-risk substance use or a substance use disorder Among the subsample with attention deficit/hyperactivity disorder as the primary diagnosis, *n* = 945,277.

Variable	Adjusted odds ratio	95% CI	*p*
Age (Ref: 15–17 years)			
0–11 years	0.392	[0.383, 0.401]	<.001
12–14 years	0.518	[0.505, 0.531]	<.001
Gender (Ref: Girls)			
Boys	1.125	[1.101, 1.149]	<.001
Race and ethnicity (Ref: white)			
Black	1.003	[0.982, 1.026]	.758
Hispanic or Latino any race	0.726	[0.705, 0.747]	<.001
Another race or ethnicity	1.976	[1.916, 2.038]	<.001
Anxiety disorder diagnosis (Ref: No)			
Yes	0.997	[0.962, 1.033]	.855
Conduct disorder diagnosis (Ref: No)			
Yes	1.386	[1.327, 1.448]	<.001
Depressive disorder diagnosis (Ref: No)			
Yes	1.361	[1.302, 1.423]	<.001
Oppositional defiant disorder diagnosis (Ref: No)			
Yes	1.379	[1.345, 1.413]	<.001
Trauma- or stressor-related disorder diagnosis (Ref: No)			
Yes	1.273	[1.229, 1.319]	<.001
Other mental health disorder diagnosis (Ref: No)			
Yes	1.671	[1.631, 1.713]	<.001

*Due to the large sample size, *p* < .001 was deemed statistically significant.

## 4 Discussion

This study examined the percentage of co-occurring high-risk substance use and SUD as well as other mental health disorders diagnoses (e.g., conduct disorder, oppositional defiant disorder, trauma- or stressor-related disorder), among youth with a primary anxiety disorder, depressive disorder, or ADHD diagnosis who received treatment from a CMHC between 2017 and 2019. In this study, approximately 1 in 20 youth with anxiety as their primary diagnosis had co-occurring high-risk substance use or a SUD, approximately 1 in 10 youth with depression as their primary diagnosis had co-occurring high-risk substance use or a SUD, and 1 in 20 youth with ADHD as their primary diagnosis had co-occurring high-risk substance use or a SUD. There were other prominent percentages of co-occurring mental health disorders across each of the three samples in this study including 6%–7% of the three groups having a co-occurring trauma- or stressor related disorder. This study also identified many differences observed in substance risk by age, gender, race and ethnicity, and the presence of other co-diagnosed mental health disorders representing key information for CMHC clinicians serving children and adolescents.

Among youth in the sample, adolescents aged 15–17 years were at the greatest risk for high-risk substance use or a SUD across the study sample. Comorbidity among adolescents with a SUD is very common, and our results are also consistent prior findings that older adolescents are at greater risk for substance use (1, 36–38). Further, older youths have been identified as being more likely to have co-occurring major depressive episodes and SUD when compared to younger youths (22). Given that the 2013–2019 mental health surveillance of youth indicates that adolescents aged 12–17 years reported alarming levels of depression, substance use, and suicidal ideation (1), the current study findings present great cause for concern, as they indicate that these high observed levels of mental and behavioral health issues, if untreated, portend graver outcomes for these youth in coming years.

Other findings in this study include boys being more likely to have co-occurring high-risk substance use or SUD compared to girls. A review paper about youths receiving primary treatment for a mental health disorder identified boys as being more likely to have any co-occurring mental health disorder when compared to girls (24). However, depending on the type of substances used and the type of mental health symptoms, other findings regarding differences between boys and girls have been mixed (22, 39). This study also identified differences of high-risk substance use or a SUD based on race and ethnicity. Across all three groups in this study, youths who were Hispanic or Latino any race had lower odds whereas individuals classified as “Another race or Ethnicity” had greater odds of having high-risk substance use or a SUD compared to White youths. Black youths with a primary depressive disorder diagnosis had higher odds of having high-risk substance use or a SUD compared to White youths with a primary depressive disorder diagnosis. However, the consistent results regarding individuals who were Hispanic or Latino of any race and individuals who were categorized as “Another race or Ethnicity” is worth further examination. Future research is needed to examine if there are state-level differences in these risks based on race and ethnicity. While the current study did not examine demographic differences by state, we identified state level differences in the co-occurrence of high-risk substance use or a SUD.

This study identified state level differences in the co-occurrence of high-risk substance use or a SUD. It is of note that, in our sample, over 95% of youth who received treatment with either an anxiety disorder, depressive disorder, or ADHD as their primary diagnosis in Alaska also had high-risk substance use or a SUD. Future research should consider how discrepancies in state-level findings may relate to policies that inhibit or expand access to mental health and substance use services [e.g., Medicaid expansion, the Children's Health Insurance Program (CHIP)]. State and regional differences in youth behavioral health also affects workforce needs, particularly given marked shortages in the mental health workforce serving children and youth (40, 41). These findings also highlight the potential for underscreening for high-risk substance use or SUD in a large proportion of facilities.

By controlling for other mental health disorder diagnoses in the logistic regression models, we consistently identified conduct, oppositional defiant, trauma- or stressor-related, and “other mental health” disorder diagnoses as associated with high-risk substance use or SUD. These findings further point to the clinical complexity of co-occurring disorder diagnoses as alongside the primary disorder diagnoses, youths with another mental health disorder may also present with high-risk substance use.

The results of this study have important implications for mental health providers who serve children and adolescents with a primary diagnosis of anxiety, depression, ADHD, high-risk substance use or SUD, and other mental health diagnoses. Expanding training or continuing education opportunities for practitioners in SUD treatment will further increase their preparedness for, favorable attitudes toward, and knowledge of working with individuals, including youths who have high-risk substance use and SUD (18, 42–45), in turn contributing to more comprehensive mental health services for individuals with co-occurring mental health disorders and SUDs. Findings from this large epidemiological study indicate the need for these trainings as slightly more than 1 in 20 of the youths in our sample had co-occurring high-risk substance use or a SUD. Despite the prevalence of substance use nationwide, there is currently a deficit of mental health providers who treat individuals with a SUD (20).

Mental health needs have been exacerbated by the COVID-19 pandemic which threatened children's mental health and doubled the prevalence of depression and anxiety symptoms (46, 47). A recent report from the U.S. Surgeon General (48) described with urgency why children and adolescent mental health is a national priority, especially in light of COVID-19 and related sequelae. This report included several recommendations related to community-based mental health resources, and highlighted the need for a skilled workforce that can effectively deliver evidence-based behavioral health interventions to children, youth, and families across a range of settings. This implies that continuing to provide community-based mental health resources and to train a skilled mental health workforce to treat anxiety, depression, ADHD, and high-risk substance use or SUDs together is critical.

### 4.1 Study limitations and future research

Despite these results having multiple implications for education, training, and clinical practice, there are also limitations that require these findings to be interpreted with caution. Firstly, there are limitations inherent to the dataset, including missing data, that some youth may have been captured multiple times in the merged dataset, and the fact that high-risk substance use was defined differently by different treatment facilities who contributed data (e.g., screening results, formal diagnosis (27–29). Because age was a categorical variable in the dataset, we were unable to utilize age as a continuous variable to observe different developmental milestones. We were also unable to utilize data regarding other gender identities other than boys and girls as the dataset includes binary male/female values for this variable. The dataset only includes diagnostic groups (e.g., anxiety disorders), and not specific diagnoses (e.g., generalized anxiety disorder); specific diagnostic criteria used for each diagnosis are also not included in the dataset. While this study focused on children and adolescents with anxiety, depression, and ADHD as primary diagnoses, future studies should examine other primary diagnoses, such as conduct disorder, oppositional defiant disorder, and trauma- or stressor-related disorder. Moreover, these studies should also analyze which type of substances (e.g., alcohol, tobacco, cannabis, sedatives, opiates, cocaine, methamphetamine) are being used and how these different types of drugs interact with anxiety, depression, and/or ADHD among children and adolescents.

## 5 Conclusion

Anxiety, depression, ADHD, and high-risk substance use and SUDs are often treated in isolation (49). Yet, given findings of a high prevalence of the co-occurrence of these diagnosed symptoms among children and adolescents (50, 51), the present study underscores the importance of integrated treatments for mental health services and substance use services in primary care and community settings, particularly CMHCs.

## Data Availability

Publicly available datasets were analyzed in this study. This data can be found here: https://www.samhsa.gov/data/data-we-collect/mh-cld-mental-health-client-level-data.
